# Does the oxidative stress play a role in the associations between outdoor air pollution and persistent asthma in adults? Findings from the EGEA study

**DOI:** 10.1186/s12940-019-0532-0

**Published:** 2019-10-29

**Authors:** Anaïs Havet, Zhen Li, Farid Zerimech, Margaux Sanchez, Valérie Siroux, Nicole Le Moual, Bert Brunekreef, Nino Künzli, Bénédicte Jacquemin, Raphaëlle Varraso, Régis Matran, Rachel Nadif

**Affiliations:** 1 INSERM U1168, VIMA: Aging and chronic diseases. Epidemiological and public health approaches, Villejuif, France; 2 Univ Versailles St-Quentin-en-Yvelines, UMRS 1168, Montigny-le-Bretonneux, France; 3 Key Laboratory of Obstetric & Gynecologic and Pediatric Diseases and Birth Defects, Ministry of Education, West China Second University Hospital, Sichuan University, Chengdu, China; 4 CHU de Lille, Laboratoire de Biochimie et Biologie Moléculaire, Pôle de Biologie Pathologie Génétique, Lille, France; 5 ISGlobal, Centre for Research in Environmental Epidemiology, Universitat Pompeu Fabra, CIBER Epidemiología y Salud Pública, Barcelona, Spain; 6 Institute for Advanced Biosciences, Centre de recherche UGA-Inserm U1209 CNRS UMR 5309, équipe d’épidémiologie environnementale, Site Santé, Allée des Alpes, Grenoble, France; 7 Institute for Risk Assessment Sciences, University Utrecht, Utrecht, the Netherlands; 8 Julius Center for Health Sciences and Primary Care, University Medical Center Utrecht, Utrecht, the Netherlands; 9 Swiss Tropical and Public Health Institute, Basel, Switzerland; 10 University of Basel, Basel, Switzerland; 11 Univ Lille et CHU de Lille, Lille, France

**Keywords:** Epidemiology, Outdoor air pollution, Persistent asthma, Oxidative stress, Fluorescent oxidation products, Mediation analysis

## Abstract

**Background:**

Evidences that oxidative stress plays a role in the associations between outdoor air pollution and asthma are growing. We aimed to study the role of plasma fluorescent oxidation products levels (FlOPs; an oxidative stress-related biomarker), as potential mediators, in the associations between outdoor air pollution and persistent asthma.

**Methods:**

Analyses were conducted in 204 adult asthmatics followed up in the French case-control and family study on asthma (EGEA; the Epidemiological study of the Genetic and Environmental factors of Asthma). Persistent asthma was defined as having current asthma at EGEA2 (baseline, 2003–2007) and EGEA3 (follow-up, 2011–2013). Exposures to nitrogen dioxide, nitrogen oxides, road traffic, particulate matter with a diameter ≤ 10 μm (PM_10_) and ≤ 2.5 μm were estimated by ESCAPE models (2009–2010), and ozone (O_3_) by IFEN models (2004). We used a mediation analysis to assess the mediated effect by FlOPs levels and the interaction between FlOPs levels and air pollution.

**Results:**

FlOPs levels increased with PM_10_ and O_3_ (adjusted β = 0.04 (95%CI 0.001–0.08), aβ = 0.04 (95%CI 0.009–0.07) per 10 μg/m^3^, respectively), and the risk of persistent asthma increased with FlOPs levels (aOR = 1.81 (95%CI 1.08–3.02)). The risk of persistent asthma decreased with exposures to NO_2_, NOx and PM_2.5_ (aOR ranging from 0.62 to 0.94), and increased with exposures to PM_10_, O_3_, O_3-summer_ and road traffic, the greater effect being observed for O_3_ (aOR = 1.78, 95% CI 0.73–4.37, per 10 μg/m^3^). Using mediation analysis, we observed a positive total effect (aOR = 2.16, 95%CI 0.70–11.9), a positive direct effect of O_3_ on persistent asthma (OR = 1.68, 95%CI 0.57–7.25), and a positive indirect effect mediated by FIOPs levels (aOR = 1.28 (95%CI 1.01–2.29)) accounting for 41% of the total effect.

**Conclusions:**

Our results add insights on the role of oxidative stress in the association between air pollution and persistent asthma.

## Background

Evidences that outdoor air pollution is associated with asthma incidence, and various asthma phenotypes as asthma control or asthma severity among asthmatics keep going up [1, 2], but the associations between outdoor air pollution and persistent asthma are very scarcely studied in adults. To our knowledge, only one study has investigated the associations between nitrogen dioxide (NO_2_) exposure, traffic road and persistent asthma, and showed that living close to a major road was associated with persistent asthma in adults followed up over eight years [3].

The underlying biological mechanisms by which outdoor air pollution may affect respiratory health include inflammatory processes, immune response modulations, genetic modifications and oxidative stress damages, which are increasingly suggested. Asthma is an oxidative stress-related disease [4], and exposures to ozone (O_3_), NO_2_ and particulate matter (PM) have been found associated with oxidative stress [5]. Studying biomarkers is a useful approach to provide new insights into the biological mechanisms that drive the disease process, to predict the development and progression of a disease and to personalize intervention strategies [6, 7]. Among the various biological markers related to oxidative stress, plasma fluorescent oxidation products (FlOPs) levels are biomarkers of damages that reflect systemic oxidative stress [8] and are of growing interest in epidemiology. In prospective studies, high plasma FlOPs levels were positively associated with the incidence of coronary heart diseases (CHD) among men without previous cardiovascular events [9], and with the risk of future CHD in women [10]. Regarding asthma, among adults from the Epidemiological study of the Genetic and Environmental factors of Asthma (EGEA), we recently reported higher plasma FlOPs levels significantly associated with asthma attacks, poor asthma control and poor lung function [11]. Overall, better understanding the underlying biological mechanisms related to asthma, and discovering novel biomarkers is the first step towards improving asthma management. To our knowledge, only two studies have investigated the associations between environmental factors and FlOPs levels. Among a population of U.S. trucking industry employees, short-term exposure to occupational PM_2.5_ was unrelated to plasma FlOPs levels [12]. In the EGEA study, occupational exposure to irritant cleaning products and to low molecular weight agents, especially highly reactive chemicals, were significantly and positively associated with higher plasma FlOPs levels in men, and an association was suggested for irritant cleaning products in women [13]. To date, the role of plasma FlOPs levels in the association between outdoor air pollution and asthma has never been studied.

We hypothesized that oxidative stress is one of the underlying biological mechanisms involved in the association between outdoor air pollution and persistent asthma. Among adults followed up in the EGEA study, we first studied the associations between outdoor air pollution (NO_2_, nitrogen oxides (NOx), particulate matter (PM), traffic load, traffic intensity, O_3_ and O_3-summer_), plasma FlOPs levels and persistent asthma. Then, according to the results, we investigated the role of plasma FlOPs levels as potential mediators in the association between outdoor air pollution and persistent asthma. We performed a mediation analysis which quantified both the mediated effect by FlOPs levels and the interaction between FlOPs levels and outdoor air pollution.

## Methods

### Study design

The EGEA is a cohort study based on an initial group of asthma cases recruited in chest clinics from five French cities (1991–1995) along with their first-degree relatives, and a group of controls (https://egeanet.vjf.inserm.fr/). The protocol and descriptive characteristics have been described previously [14, 15], and inclusion criteria used to define asthmatic cases and controls were described in Additional file 1. A 12-year follow-up of the initial cohort was conducted between 2003 and 2007 (EGEA2) [16], and 1571 adults aged ≥16 years had a complete examination. As a follow-up study of EGEA2, the third survey (EGEA3, 2011–2013, *n* = 1558) was conducted using self-completed questionnaire only**.** The EGEA collection was certified ISO 9001 since 2006 to 2018 [17]. Ethical approval was obtained from the relevant institutional review board committees (Cochin Port-Royal Hospital and Necker-Enfants Malades Hospital, Paris). All participants signed a written informed consent.

The analyses included 204 adults with current asthma at EGEA2, followed up at EGEA3, and with data on outdoor air pollution and plasma FlOPs levels (Additional file 2: Figure S1). In comparison to participants not included in the analyses, participants included had lower body mass index, lived longer at the same residential address and had lower exposure to NO_2_, PM_2.5_, O_3_ and O_3-summer_ (all *P*-value≤0. 05, Additional file 1: Table S1). No differences were found regarding other variables.

### Definition of persistent asthma

At EGEA2, the participants with ever asthma answered positively to at least one of the two following questions: “*Have you ever had attacks of breathlessness at rest with wheezing?” or “Have you ever had asthma attacks?*”, or were recruited as asthmatic cases at EGEA1. Among participants with ever asthma, those with current asthma reported asthma attacks or the use of asthma medication in the past twelve months. Participants with persistent asthma had current asthma at both EGEA2 and EGEA3, and those with remittent asthma had current asthma only at EGEA2. Participants with remittent asthma were used as reference (see Additional file 1 and for further respiratory outcomes definitions).

### Exposure assessment

Available air pollution data from ESCAPE (European Study of Cohorts for Air Pollution Effects) were NO_2_, NOx and particulate matter with a diameter ≤ 10 and ≤ 2.5 μm (PM_10_ and PM_2.5_), and those from IFEN (French Institute for the Environment) were O_3_ and O_3-summer_. Outdoor air pollution exposures were assigned to each participant’s residential address.

Annual air pollution levels of NO_2_ and particulate matter were derived from ESCAPE standardised models (www.escapeproject.eu). Briefly, the ESCAPE monitoring campaigns took place between 2009 and 2010, including 40 measurement sites for NO_2_ and NO_x_ in Paris, Lyon, Grenoble and Marseille, and 20 particulate matter measurement sites in Paris and Grenoble. Land-use regression (LUR) models were developed and two indicators of road traffic exposure were also calculated. Traffic intensity on nearest road was defined as the number of motor vehicles circulating per day on the nearest road to the participant’s home and was expressed in vehicles per day. Total traffic load was defined as the traffic load on all major roads based around a buffer of 100 m from the participant’s home and was expressed by traffic intensity multiplied by road length. Back-extrapolation was used to transfer the current LUR models (2009–2010) to earlier years (2003–2007, EGEA2) (see Additional file 1 for more details). In our study, the estimation of outdoor air pollution by ESCAPE took place after EGEA2, and accordingly we also analysed the back-extrapolated pollution estimates in order to obtain a better temporality between outdoor air pollution and plasma collection. Back-extrapolated pollution data were available for NO_2_ and NO_x_ in all cities, and for PM_10_ in Paris. The spatial resolution was 50 m × 50 m. In order to supplement the ESCAPE data set, we used O_3_ and O_3-summer_ exposures from the IFEN (see Additional file 1 for more details). The O_3_ estimate was the yearly mean ozone level in 2004 for each participant at the residential address and derived from a geo-statistical model as described previously [18]. The O_3-summer_ exposure was assessed from the monthly means from April to September. The spatial resolution was 4 km × 4 km.

### Measurement of plasma FlOPs levels

Plasma samples were collected in EGEA2 between 2003 and 2006 and stored immediately at − 80 °C during 5.0 to 8.0 years until FlOPs measurements. Plasma FlOPs levels were measured as previously described [8, 13] (Additional file 1). Briefly, plasma was extracted into a mixture of ethanol/ether (3/1 v/v) and measured using a spectrofluorimeter (360 nm excitation wavelength, 430 nm emission wavelength). Fluorescence was expressed as a unit of relative fluorescence intensity (RFU/mL) of plasma.

### Statistical methods

Due to their skewed distribution, plasma FlOPs levels were log_10_-transformed. Due to the familial dependence of the data, multivariate analyses (except mediation analyses) took into account dependence between observations. Linear regression models and logistic regression models with random effects on center and familial dependence were used to study the associations between outdoor air pollution with plasma FlOPs levels, and between outdoor air pollution and persistent asthma, respectively. To control a potential effect of short-term exposure to O_3_ in the associations between O_3_ with plasma FlOPs levels and persistent asthma, further adjustment for the season of plasma collection (EGEA2) was conducted. Logistic regression models using generalized estimated equations (GEEs) on familial dependence were performed to study associations between plasma FlOPs levels and persistent asthma. To study only the road traffic effect, estimates of associations between road traffic with persistent asthma or plasma FlOPs levels were also adjusted for background NO_2_. To obtain a better temporality between outdoor air pollution assessed by ESCAPE and plasma collection, analyses with back-extrapolated pollution estimates were also performed.

**Fig. 1:**
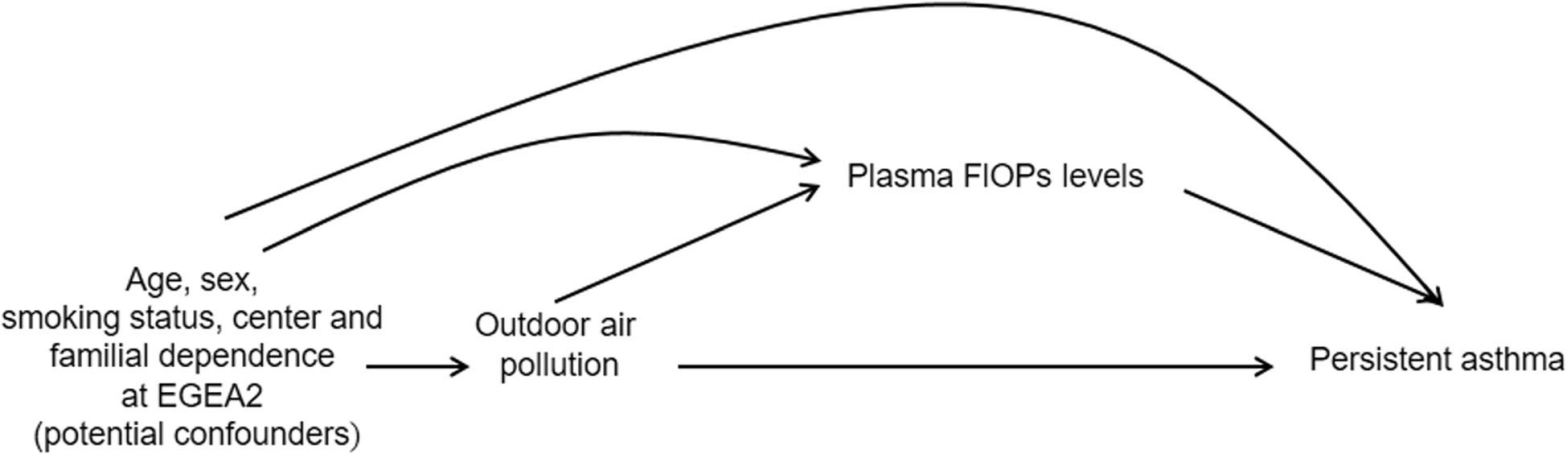
Direct acyclic graph of the proposed mediation model. FlOPs: fluorescent oxidation products

We used a direct acyclic graph to represent our mediation model (Fig. 1), and conducted mediation analysis based on a counterfactual approach by using the CAUSALMED procedure [19, 20] (see Additional file 1). Mediation analysis was performed for air pollutants associated with both persistent asthma and plasma FlOPs levels, regardless of the significance of the association between air pollutant and persistent asthma. Models did not include random effects on center and familial dependence. The four-way decomposition was used to investigate the proportions of total effect that were attributable to the controlled direct effect, to mediation (the pure indirect effect), to interaction (the reference interaction between pollutant and plasma FlOPs levels) and to both mediation and interaction (the mediated interaction) [21]. Percentages mediated and due to interaction were given. All these components were defined in Additional file 1.

All estimates were adjusted for age (continuous), sex and smoking status (never-, ex- or current smokers). We defined never-smoker as a participant who have never smoked in their life, ex-smoker as a participant who quit smoking for at least 4 weeks at EGEA2 and current smoker as a participant who was smoking at least one cigarette a day for more than 1 year at EGEA2. The results are expressed for an increase of 20 μg/m^3^ of NO_x_, 10 μg/m^3^ of NO_2_, PM_10_, O_3_ and O_3-summer_ and 5 μg/m^3^ of PM_2.5._ The results of traffic load are expressed for 4 million vehicles multiplied by meters per day on major roads within a 100 m buffer, and those of traffic intensity for an increase of 5000 vehicles per day on major roads. We performed sensitivity analyses by excluding participants who lived at the same address for < 1 year. Statistical analyses were performed using SAS version 9.4 (SAS Institute, Cary, NC, USA).

## Results

At EGEA2, the mean age of the 204 adults was 39 years, 48% were men, 24% were current smoker, 79% had persistent asthma, and the geometric mean (interquartile range) of plasma FlOPs levels was 92.0 (79.5–104.7) RFU/mL (Table 1). In comparison to participants with remittent asthma, participants with persistent asthma had more often poor controlled asthma and an asthma symptom score > 2, reported more often the use of inhaled corticosteroids, and had higher plasma FlOPs levels (all *P* values≤0.02, Table 1). Plasma FlOPs levels increased with age (p-trend< 0.0001, Additional file 1: Table S2), were lower in never smokers than smokers (*p* = 0.02, Additional file 1: Table S2). No significant association was found between plasma FlOPs levels and other variables (Additional file 1: Table S2).

**Table 1:** Description of participants with current asthma at baseline (EGEA2) according to change in current asthma between EGEA2 to EGEA3

	All participants	Participants with remittent asthma	Participants with persistent asthma	*P*-value
N	204	43	161	
Age (years), mean ± SD	39.3 ± 16.9	36.7 ± 15.3	40.0 ± 17.3	0.26
Male, n (%)	99 (48.5)	16 (37.2)	83 (51.6)	0.09
Smoking status, n (%)				
Never-smoker	109 (53.4)	22 (51.2)	87 (54.0)	0.52
Ex-smoker	46 (22.6)	8 (18.6)	38 (23.6)	
Current smoker	49 (24.0)	13 (30.2)	36 (22.4)	
BMI (kg/m^2^), mean ± SD	*n* = 203	23.4 ± 3.99	*n* = 160	0.40
	23.8 ± 3.84		23.9 ± 3.81	
Socioprofessional category, n (%)				
Unemployed	32 (15.7)	5 (11.6)	27 (16.8)	0.37
Manager	64 (31.4)	12 (27.9)	52 (32.3)	
Technician	88 (43.1)	19 (44.2)	69 (42.8)	
Manual worker	20 (9.8)	7 (16.3)	13 (8.1)	
Asthma onset years, mean ± SD	*N* = 195	*N* = 39	*N* = 156	0.40
	14.8 ± 14.7	16.6 ± 14.6	14.4 ± 14.7	
Poor controlled asthma^a^, n (%)	*N* = 190	*N* = 38	*N* = 152	0.007
	102 (53.7)	13 (34.2)	89 (58.6)	
Use of inhaled corticosteroids, n (%)	*N* = 200	*N* = 41	*N* = 159	0.009
	132 (66.0)	20 (48.8)	112 (70.4)	
Symptom score of asthma^b^, n (%)	*N* = 199	*N* = 40	N = 159	
0	20 (10.0)	9 (22.5)	11 (6.9)	0.005
1–2	30 (15.1)	8 (20.0)	22 (13.8)	
>2	149 (74.9)	23 (57.5)	126 (79.3)	
Plasma FlOPs levels (RFU/mL), GM (q1-q3)	92.0 (79.5–104.7)	85.6 (76.0–96.5)	93.8 (79.9–104.8)	0.02
Residence time (years), mean ± SD	11.7 ± 10.2 (0–42)	10.3 ± 8.90 (0–31)	12.1 ± 10.5 (0–42)	0.31
Air pollutant levels and traffic metrics^c^, mean ± SD				
NO_2_	27.9 ± 13.9	29.7 ± 15.3	27.4 ± 13.5	0.34
NO_X_	49.7 ± 32.7	53.2 ± 38.4	48.8 ± 31.0	0.44
PM_10_	25.0 ± 4.07	25.1 ± 3.71	24.9 ± 4.18	0.88
PM_2.5_	15.0 ± 2.04	15.5 ± 1.96	14.9 ± 2.05	0.20
Traffic load	1,755,353 ± 4,718,630	1,672,062 ± 4,007,561	1,777,599 ± 4,902,148	0.90
Traffic intensity	7307 ± 13,109	7027 ± 11,360	7382 ± 13,569	0.87
O_3_	43.9 ± 4.62	43.1 ± 4.08	44.2 ± 4.74	0.15
O_3-summer_	65.4 ± 6.20	64.7 ± 6.41	65.6 ± 6.14	0.38

*SD* standard deviation, *BMI* body mass index, *FlOPs* fluorescent oxidation products, *GM* geometric mean; q1-q3: 25th and 75th percentiles of the GM

^a^Defined according to GINA 2015 guidelines and from participants with partly controlled asthma or uncontrolled asthma (see Additional file 1)

^**b**^More details in Additional file 1

^c^Pollutant levels in μg/m^3^, traffic load in vehicles/day x meters, and traffic intensity in vehicles/day

### Associations between outdoor air pollution, plasma FlOPs levels and persistent asthma

Among all pollutants studied, plasma FlOPs levels increased by 1 RFU/mL with PM_10_ and O_3_ exposures (adjusted (a)β = 0.04, 95% CI 0.001–0.08, *p* = 0.03 and aβ = 0.04, 95% CI 0.009–0.07, *p* = 0.02 for an increase of 10 μg/m^3^ of O_3_ and PM_10_, respectively, Table 2). The results were similar after excluding participants who lived at the same address for less than 1 year. The association between PM_10_ and plasma FlOPs levels did not remain significant with back-extrapolated data (aβ = 0.03, 95% CI -0.01–0.07, *p* = 0.22 for an increase of 10 μg/m^3^ of PM_10_, Table 2). The risk of persistent asthma decreased not significantly with exposure to NO_2_, NOx and PM_2.5_ (aOR ranging from 0.62 to 0.94), and increased not significantly with exposure to PM_10_, O_3_, O_3-summer_ and road traffic, the greater effect being observed for O_3_ (aOR = 1.78, 95% CI 0.73–4.37, for an increase of 10 μg/m^3^ of O_3_, model 2, Additional file 1: Table S3). Results were similar after excluding participants who lived at the same address for less than 1 year (Table 2 and Additional file 1: Table S3). Further adjustment for season of plasma collection did not change the results with O_3_ (data not shown). The risk of persistent asthma increased with plasma FlOPs levels (unadjusted OR = 1.68, 95% CI 1.04–2.70, *p* = 0.03 for an increase of 1 interquartile range of FlOPs levels), and this association remained consistent after adjustment for age, sex and smoking status (aOR = 1.81, 95% CI 1.08–3.02, p = 0.02) for an increase of 1 interquartile range of FlOPs levels).

**Table 2:** Associations between outdoor air pollution and plasma FlOPs levels

		NO_2_	NO_x_	PM_10_^a^	PM_2.5_^a^	Total traffic load on major roads in a 100-m buffer of the home^b^	Traffic intensity at the road nearest to a participant’s home^b^	O_3_	O_3-sumer_
Model 1	n β crude (95% CI) P	204 0.007 (− 0.004,0.02) 0.19	204 0.006 (− 0.003,0.02) 0.15	146 0.04 (0.001,0.08) 0.04	146 0.03 (− 0.009,0.07) 0.15	204 0.008 (− 0.005,0.02) 0.24	204 0.0004 (− 0.005,0.006) 0.88	204 0.03 (− 0.004,0.06) 0.06	204 0.02 (− 0.03,0.07) 0.36
Model 2	n β adjusted (95% CI) P	204 0.007 (− 0.003,0.02) 0.18	204 0.007 (− 0.002,0.02) 0.16	146 0.04 (0.001,0.08) 0.03	146 0.03 (− 0.008,0.07) 0.18	204 0.006 (− 0.006,0.02) 0.30	204 0.0002 (− 0.005,0.006) 0.94	204 0.04 (0.009,0.07) 0.02	204 0.02 (− 0.02,0.06) 0.45
Model 3	n β adjusted (95% CI) P	204 0.007 (− 0.002,0.02) 0.14	204 0.006 (− 0.002,0.01) 0.12	72 0.03 (− 0.01,0.07) 0.22^c^					
Model 4	n β adjusted (95% CI) P	186 0.009 (− 0.003,0.02) 0.14	186 0.008 (− 0.002,0.02) 0.14	133 0.05 (0.006,0.09) 0.04	133 0.02 (− 0.02,0.06) 0.24	186 0.003 (− 0.01,0.02) 0.63	186 0.0005 (− 0.005,0.006) 0.86	186 0.05 (0.01,0.08) 0.01	186 0.02 (− 0.03,0.07) 0.37

The linear regression models were conducted with random effects on familial dependence and center. FlOPs levels were log_10_-transformed. NO_2_, nitrogen dioxide; NO_x_, nitrogen oxides; PM_10_, particulate matter with a diameter ≤ 10 μm; PM_2.5_, particulate matter with a diameter ≤ 5 μm. Results are expressed per 20 μg/m^3^ increase of NO_x_ exposure, per 10 μg/m^3^ increase of NO_2_, PM_10_ O_3_ and O_3-summer_ exposures, per 5 μg/m^3^ increase of PM_2.5_ exposure, per 4 million vehicles x meters per day increase of total traffic load, per 5000 vehicles per day increase of traffic intensity. Model 1: unadjusted. Model 2: adjusted for age, sex and smoking status. Model 3: with back-extrapolated pollution and adjusted for age, sex and smoking status. Model 4: after excluding the participants living at the same residential address <1 year

^a^Not back-extrapolated PM were only estimated in Paris and in Grenoble, and back extrapolated PM_10_ only in Paris

^b^Estimates were also adjusted for background NO_2_

^c^The linear regression model was only conducted with random effects on familial dependence because back-extrapolated PM_10_ were estimated only in Paris

### Ozone, FlOPs levels and persistent asthma: mediation analysis

Results were summarized in Table 3. We observed a positive total effect (OR_TE_ = 2.16, 95% CI 0.70–11.9, *P* > 0.05), a positive natural direct effect of O_3_ on persistent asthma (OR_CDE_ = 1.68, 95% CI 0.57–7.25, P > 0.05), and a positive natural indirect effect mediated by plasma FlOPs levels (OR_NIE_ = 1.28, 95% CI 1.01–2.29, *P* = 0.04). Further decomposition of total effect showed that 50.3% of total effect was due to control direct effect, 8.6% due to the reference interaction, 26.5% due to mediated interaction and 14.6% due to the pure indirect effect. The percentage of total effect mediated by plasma FlOPs levels was 41.1% (26.5% + 14.6%). The percentage of total effect due to interaction was 35.1% (8.6% + 26.5%). These results were quite similar after excluding participants who lived at the same address for < 1 year (Additional file 1: Table S4). We also found that the controlled direct effect increased with plasma FlOPs levels (Additional file 1: Table S5).

**Table 3:** Results of mediation analysis between O_3_, persistent asthma and plasma FlOPs levels using the CAUSALMED procedure (*n* = 204)

OR	Estimate	95% CI
Odds Ratio Total Effect	2.16	0.70–11.9
Odds Ratio Natural Direct Effect	1.68	0.57–7.25
Odds Ratio Natural Indirect Effect	1.28	1.01–2.29
Decomposition of the total effect		
Four-way	Percent	
Controlled direct	50.3	
Reference interaction	8.6	
Mediated interaction	26.5	
Pure indirect	14.6	

Models were adjusted for age, sex and smoking habits. The exposure was O_3_. The mediators were plasma FlOPs levels. The outcome was persistent asthma

## Discussion

We studied and quantified the role of plasma FlOPs levels in the association between outdoor air pollution and persistent asthma. We found that plasma FlOPs levels increased with PM_10_ and O_3_ exposures, and the risk of persistent asthma increased with plasma FlOPs levels. We also found that the risk of persistent asthma increased with O_3_ exposure, but not significantly. We therefore performed mediation analysis to investigate the role of plasma FlOPs levels in the association between O_3_ exposure and persistent asthma. We found positive indirect effect mediated by plasma FIOPs levels accounting for 41% of the total effect, and O_3_ effect on persistent asthma increased with plasma FlOPs levels. Due to the limited sample size and borderline significant findings, and to the other biomarkers related to oxidative stress potentially involved in this association, the results need to be interpreted with caution.

To our knowledge, this study is the first one investigating the associations between outdoor air pollution and plasma FlOPs levels. FlOPs levels are biomarkers of damages related to oxidative stress reflecting a mixture of oxidation products from lipids, proteins and DNA [6]. These biomarkers are of growing interest in epidemiological studies because they are stable, easily measurable and applicable in large-scale human studies [9]. We found that plasma FlOPs levels increased with long-term exposure to O_3_, an irritant gas with a strong oxidative potential [5]. To date, no study has investigated the associations between O_3_ exposure and plasma FlOPs levels, and a recent review reported positive and significant associations between short-term exposure to O_3_ and 8-isoprostane, another biomarker related to oxidative stress [22]. We did not have data to properly assess by which extent acute exposure to O_3_ (over the past hours/days) could have biased our results. Nevertheless, the adjustment for the season of plasma collection did no change the results. As plasma FlOPs levels are biomarkers of damages that reflect cumulative oxidative stress, we hypothesized to find associations with long-term exposure to air pollution rather than with short-term air pollution. Further studies are needed to differentiate the effects of short-term and long-term exposure to air pollution, and in particular O_3_, on plasma FlOPs levels. We also found that plasma FlOPs levels increased with non-back extrapolated PM_10._ The way and the strength of the association with back-extrapolated PM_10_ were the same, but the association was not significant due to the small effective. Previously, we found that 8-isoprostane in exhaled breath condensate, a matrix close to the lungs, increased significantly with PM_2.5_ exposure in the EGEA study [23]. 8-isoprostane is a biomarker of damages related to oxidative stress, and a specific product of lipid peroxidation. In the present analysis, plasma FlOPs levels increased with PM_2.5_ exposure but the association was not significant. Fine and ultrafine particulates are known to be more harmful by penetrating deeper into the lungs and inducing damages due to oxidative stress both at the airways and systemic compartment [24]. The discrepancies in the results could be partly explained by difference in the sample sizes, in the composition and concentration of the particulates, and by difference in underlying mechanism related to the studied biomarker. In a previous work conducted among participants without asthma in the EGEA study, occupational exposure to irritant cleaning/disinfecting agents increased plasma FlOPs levels [13]. Overall, all these results underlined that occupational exposure to irritants, and outdoor air pollution exposure, especially exposure to O_3_ and PM_10_, were associated with higher plasma FlOPs levels, and outdoor air pollution increased oxidative stress at both lung and systemic level.

We studied persistent asthma in association with O_3_, PM and plasma FlOPs levels. Asthma is a chronic and heterogeneous disease defined by various overlapping phenotypes, including the phenotype “persistent asthma” [25]. Although persistent asthma is still under-studied in epidemiology, it reflects the activity and the evolution of asthma over time. Most associations between pollution and persistent asthma were close to 1; the risk of persistent asthma decreased with PM_2.5_ and increased with O_3_ and O_3-summer_ exposures. The unexpected result observed for PM_2.5_ may be partly due to the lack of back-extrapolated data leading to an inverse temporality between PM_2.5_ and persistent asthma, or to random effect or residual bias. To date, only one study investigated the associations between exposure to outdoor air pollution and with persistent asthma in adults, and showed that living within 200 m of a major road was associated with persistent asthma in middle-aged Tasmanian participants [3]. Like us, the authors defined “current asthma” as “*any episode of asthma or use of asthma medication during the last 12 months”.* We also found that the risk of persistent asthma increased with plasma FlOPs levels. Our definition of “current asthma” included the report of asthma attack and the use of respiratory treatment in the last twelve months, and interestingly in adults of the EGEA study, asthma attacks, any asthma treatment and use of inhaled corticosteroids in the past 12 months were positively associated with plasma FlOPs levels [11]. Interestingly, leukocyte telomere length, which reflects oxidative-stress damages to DNA [26], was shorter in participants who had persistent asthma from childhood into adult as compared to those who had adolescent or adult-onset asthma [27]. From a larger sample size, it would be now interesting to study the associations between outdoor air pollution, plasma FlOPs levels and asthma incidence.

We acknowledge that performing a mediation analysis despite the non-significant association between O_3_ and persistent asthma may open a debate. We based our decision on the biological hypothesis that oxidative stress is one mechanism by which outdoor air pollution affects respiratory health. Although the association between O_3_ and persistent asthma was non-significant, the strength of the association between O_3_ and persistent asthma, and previous results obtained in the EGEA study guided our decision. Indeed, O_3_ was associated with severe asthma [18], uncontrolled asthma [28] and current asthma [23] in adults.

The CAUSALMED procedure is a recent tool available in SAS software to estimate causal mediation effects from observational data [19]. This procedure is advisable without prior knowledge about the lack of the interaction [29]. In mediation analysis, the random effects on center and familial dependence were not taken into in models, explaining the differences of results between the association between O_3_ and persistent asthma studied outside the mediation analysis and the direct effect. Beyond direct and indirect effects, the four-way decomposition shed insights into the role of both mediation and interaction in the associations between O_3_ and plasma FlOPs levels with persistent asthma. Overall, our mediation analysis may suggest a not negligible effect of O_3_ on persistent asthma through plasma FlOPs levels, and more precisely that effect of O_3_ on persistent asthma increased with plasma FlOPs levels. Due to the small sample size, the estimates from mediation analysis may be imprecise and the results should therefore be interpreted with caution. Further studies, with a larger study sample, using a model including other biomarkers related to oxidative stress or biomarkers related to other pathways, as well as the interrelations between these biomarkers, would be helpful to better understand the underlying biological mechanisms between outdoor air pollution and asthma.

## Conclusion

For the first time in adults, we found that plasma FlOPs levels increased with O_3_ and PM_10_ exposures, and the risk of persistent asthma increased with plasma FlOPs levels. Overall, our results add insights into the potential role of plasma FlOPs levels in the association between O_3_ and persistent asthma, and add new evidence on the role of oxidative stress in the association between outdoor air pollution and asthma.

## Supplementary information

**Additional file 1: Table S1.** Description of participants included and not included in analyses. **Table S2.** Associations between plasma FlOPs levels and characteristics of participants. **Table S3.** Associations between outdoor air pollution and persistent asthma. **Table S4.** Results of mediation analysis using the CAUSALMED procedure among participants who lived at the same address for > 1 year (*n* = 186). **Table S5.** Controlled direct effect according to quantiles of plasma FlOPs levels (*n* = 204).

**Additional file 2: Figure S1.** Flow chart of the studied population.

## Data Availability

Due to third party restrictions, EGEA data are not publicly available. Please see the following URL for more information: https://egeanet.vjf.inserm.fr/index.php/en/contacts-en. Interested researchers should contact egea. cohorte@inserm.fr with further questions regarding data access.

## Abbreviations

CI: confidence interval
EGEA: Epidemiological study of the Genetic and Environmental factors of Asthma
ESCAPE: European Study of Cohorts for Air Pollution Effects
FlOPs: fluorescent oxidation products
IFEN: French Institute of Environment
NO_2_: nitrogen dioxide
NOx: nitrogen oxides
O_3_: ozone
O_3-sumer_: summer ozone
OR: odds ratio
PM_10_: particulate matter with a diameter ≤ 10 μm
PM_2.5_: particulate matter with a diameter ≤ 2.5 μm
