# Supplementary material for: Does the oxidative stress play a role in the associations between outdoor air pollution and persistent asthma in adults? Findings from the EGEA study
Source: Environ Health. 2019 Oct 29;18:90. doi: 10.1186/s12940-019-0532-0 (PMC6819357; doi:10.1186/s12940-019-0532-0)
Supplement: Supplementary file 1 — Additional file 1: Table S1. Description of participants included and not included in analyses. Table S2. Associations between plasma FlOPs levels and characteristics of participants. Table S3. Associations between outdoor air pollution and persistent asthma. Table S4. Results of mediation analysis using the CAUSALMED procedure among participants who lived at the same address for > 1 year (n = 186). Table S5. Controlled direct effect according to quantiles of plasma FlOPs levels (n = 204). [file 12940_2019_532_MOESM1_ESM.doc]

**Additional files**

**Does the oxidative stress play a role in the associations between outdoor air pollution and persistent asthma in adults? Findings from the EGEA study.**

Anaïs Havet^1,2*^, Zhen Li^3^, Farid Zerimech^4^, Margaux Sanchez^5^, Valérie Siroux^6^, Nicole Le Moual^1,2^, Bert Brunekreef^7,8^, Nino Künzli^9,10^, Bénédicte Jacquemin^1,2^, Raphaëlle Varraso^1,2^, Régis Matran^1*^, Rachel Nadif^1,2^

*Corresponding author. INSERM UMR-S 1168 VIMA: Aging and chronic diseases. Epidemiological and public health approaches. 16 avenue Paul Vaillant Couturier, F-94807 VILLEJUIF Cedex. E-mail: [anais.havet@inserm.fr](mailto:anais.havet@inserm.fr)

RM and RN contributed equally.

**Methods**

**Study population**

Data used for the analyses were collected in the framework of the EGEA study (<https://egeanet.vjf.inserm.fr/>). EGEA is a French cohort study based on an initial group of asthma cases and their first-degree relatives, and controls (first survey EGEA1, between 1991 and 1995, n=2047). The protocol and descriptive characteristics have been described previously [1,2].

A 12-year follow-up of the initial cohort was conducted between 2003 and 2007 (EGEA2) [3]. Among the alive cohort (n=2002), 92% (n=1845) completed a short self-administered questionnaire, and among them 1571 adults aged ≥16 years had a complete examination. All subjects responded to a questionnaire based on international standardized tools to diagnose asthma and to determine respiratory and allergic symptoms, treatments, and environmental exposures.

As a follow-up study of EGEA2, the third survey (EGEA3) was conducted in 2011 using self-completed questionnaire and 1558 questionnaires were returned.

**Respiratory phenotypes**

Inclusion criteria used to define asthma cases at EGEA1 were based on self-reported positive responses to four questions from the validated and standardized British Medical Research Council, European Coal and Steel Community, American Thoracic Society (ATS) and European Community Respiratory Health Survey (ECRHS) questionnaires: “*Have you ever had attacks of breathlessness at rest with wheezing?”*, *“Have you ever had asthma attacks?*”, “*Was this diagnosis confirmed by a physician?*” and “*Have you had an asthma attack in the last 12 months?*”, or a positive response to at least two questions and a positive review of the medical records. Asthma in first-degree relatives of asthma cases was defined as a positive answer to at least one of the first two questions [4,5]. At EGEA2, the participants with ever asthma answered positively to at least one of the two following questions: “*Have you ever had attacks of breathlessness at rest with wheezing?” or “Have you ever had asthma attacks?*”, or were recruited as asthmatic cases at EGEA1.

Asthma control has been assessed over 3 month period, using responses to EGEA2 survey questions to approximate the Global Initiative for Asthma 2015 definition as closely as possible and as previously used [6].

We used the asthma symptom score defined by Sunyer *et al*. (2007) [7]. Ranging from 0 to 5, the score is based on the number of respiratory symptoms during the past 12 months reported in the questionnaire: breathless while wheezing, woken up with chest tightness, attack of shortness of breath at rest, attack of shortness of breath after exercise and woken by attack of shortness of breath.

**Exposure assessment**

The ESCAPE assessment used land-use regression (LUR) models, developed to explain the spatial variation of pollutant exposures within each city. Predictor variables were calculated using the site coordinates and digital data thanks to geographical information system [8]. Two indicators of road traffic were also calculated: traffic intensity on the nearest road (vehicles per day), and total traffic load on all major roads within 100-buffer (intensity multiplied by road length). The spatial resolution is 50 meters. In the ESCAPE project, models to estimate back-extrapolated levels of PM and NO_x_ in previous years were based on historic data on land use and road networks, and background levels of PM and NO_x_. The back-extrapolated concentration was estimated by multiplying the modeled ESCAPE annual mean concentration by the ratio between average annual concentrations as derived from the routine monitoring site(s) for the period in the past and for the ESCAPE measurement period time: Cextrapolated-ratio = CESCAPE * Ratioroutine, with Ratioroutine = Croutine-baseline / Croutine-ESCAPE [9].

The IFEN assessment used the geostatistical interpolation techniques (kriging-like techniques) to estimate air pollution at unsampled locations, taking account the spatial structure of each pollutant. Interpolation was done for pollution estimates coming from background monitoring stations on a 4kmx4km grid covering France. Land cover was integrated to the interpolation process as well as specific cofactors correlated with the pollutants such as altitude and north-south concentration gradient for O_3_.

**Measurement of plasma FlOPs**

Plasma FlOPs levels were measured as previously described [10,11]. Briefly, plasma was extracted into a mixture of ethanol/ether (3/1 v/v) and measured using a spectrofluorimeter (360 nm excitation wavelength, 430 nm emission wavelength). Fluorescence was expressed as a unit of relative fluorescence intensity (RFU)·mL−1 of plasma. Each sample was replicated. The intra-assay coefficient of variation (CV) for FlOPs was less than 20%. The dosages for which the CV were ≥ 20 % have been removed of analysis. In the literature, FlOPs are described as stable biomarkers over time (11) and in the EGEA study, no significant association was found between storage time and plasma FlOPs levels (regression coefficient= 0.002, p=0.08).

**Mediation analysis**

We applied the method proposed by Valeri and VanderWeele [12] by using the CAUSALMED procedure in the SAS software. Based on the counterfactual framework, the CAUSALMED procedure implements the regression adjustment method to estimate causal mediation effects [13]. The advantage of the CAUSALMED procedure was that it is suitable to linear and nonlinear models, with or without interaction effects, and for continuous or binary outcomes, exposures and mediators. We assumed that the following conditions were satisfied: no unmeasured confounders for the associations between 1) O_3_–FlOPs levels, 2) O_3_–persistent asthma, 3) FlOPs levels–persistent asthma, and 4) no FlOPs levels–persistent asthma confounders affected by outdoor air pollution. We used bootstrap resampling to compute standard errors and confidence intervals. The four-way decomposition was used to investigate the proportions of total effect (TE) that were attributable to controlled direct effect (CDE), to mediation (the pure indirect effect or PIE), to interaction (the reference interaction or INT_ref_) and to both mediation and interaction (the mediated interaction or INT_med_) [14]. The TE is equal to the sum of natural direct effect (NDE) and natural indirect effect (NIE), with NDE = CDE + INT_ref_ and NIE = PIE + INT_med_. In other words, the CDE corresponded to the effect of O_3_ on persistent asthma regardless of mediator. The PIE corresponded to the effect of FlOPs levels on persistent asthma in absence of O_3_, even if O_3_ was necessary for the FlOPs levels to be present. The INT_ref_ corresponded to the effect of O_3_ on persistent asthma in presence of FLOPs levels. The INT_med_ corresponded to the effect of O_3_ on FlOPs levels, plus to the effect of O_3_ on persistent asthma in presence of FLOPs levels. The percentage mediated corresponded to the percentage of TE that is mediated by FlOPs levels, and the percentage attributed to interaction corresponded to the percentage of total effect due to the interaction between O_3_ and FlOPs levels. Since the percentage of the total effect due to the interaction between O_3_ and FlOPs levels was not negligible, we also quantified the control direct effect at different levels of plasma FlOPs by using the EVALUATE option, as described in the paper by Yiu-Fai Yung *et al*. (2018) [15].

**References**

1. Kauffmann F, Dizier MH. EGEA (Epidemiological study on the Genetics and Environment of Asthma, bronchial hyperresponsiveness and atopy)--design issues. EGEA Co-operative Group. Clin. Exp. Allergy J. Br. Soc. Allergy Clin. Immunol. 1995;25 Suppl 2:19–22

2. Kauffmann F, Dizier MH, Annesi-Maesano I, Bousquet J, Charpin D, Demenais F, et al. EGEA (Epidemiological study on the Genetics and Environment of Asthma, bronchial hyperresponsiveness and atopy) – descriptive characteristics. Clin Exp Allergy 1999;29 Suppl 4:17–21.

3. Bouzigon E, Nadif R, Le Moual N, Dizier MH, Aschard H, Boudier A, et al. Facteurs génétiques et environnementaux de l’asthme et de l’allergie : synthèse des résultats de l’étude EGEA. Rev. Mal. Respir. 2015;32(8):822–840.

4. Burney PGJ, Luczynska C, Chinn S, Jarvis D. The European Community Respiratory Health Survey. Eur. Respir. J. 1994; 7(5): 954–960.

5. Kauffmann F, Dizier M-H, Pin I, Patye E, Gormand F, Vervloet D, et al. Epidemiologic Study of the Genetics and Environment of Asthma, Bronchial Hyperresponsiveness, and Atopy. Am. J. Respir. Crit. Care Med. 1997; 156(4 Pt 2): S123–S129.

6. Siroux V, Boudier A, Dolgopoloff M, Chanoine S, Bousquet J, Gormand F, et al. Forced midexpiratory flow between 25 % and 75 % of forced vital capacity is associated with long-term persistence of asthma and poor asthma outcomes. J. Allergy Clin. Immunol. 2016; 137(6): 1709–1716.

7. Sunyer J, Pekkanen J, Garcia-Esteban R, Svanes C, Künzli N, Janson C, et al. Asthma score : predictive ability and risk factors. Allergy 2007; 62(2): 142–148.

8. Eeftens M, Beelen R, De Hoogh K, Bellander T, Cesaroni G, Cirach M, et al. Development of land use regression models for PM_2.5_, PM_2.5_ absorbance, PM_10_ and PMcoarse in 20 European study areas; Results of the ESCAPE project. Environ. Sci. Technol. 2012; 46(20): 11195–11205.

9. Beelen R, Raaschou-Nielsen O, Stafoggia M, Andersen ZJ, Weinmayr G, Hoffmann B, et al. Effects of long-term exposure to air pollution on natural-cause mortality: An analysis of 22 European cohorts within the multicentre ESCAPE project. Lancet 2014; 383(9919): 785–795.

10. Dumas O, Matran R, Zerimech F, Decoster B, Huyvaert H, Ahmed I, et al. Occupational exposures and fluorescent oxidation products in 723 adults of the EGEA study. Eur. Respir. J. 2015;46(1):258–261.

11. Wu T, Willett WC, Rifai N, Rimm EB. Plasma fluorescent oxidation products as potential markers of oxidative stress for epidemiologic studies. Am. J. Epidemiol. 2007;166(5):552–560.

12. Valeri L, Vanderweele TJ. Mediation Analysis Allowing for Exposure – Mediator Interactions and Causal Interpretation : Theoretical Assumptions and Implementation With SAS and SPSS Macros. Psychological Methods 2013;18(2):137–150. 2

13. SAS/STAT® 14.3 User’s Guide The CAUSALMED Procedure. <https://support.sas.com/documentation/onlinedoc/stat/143/causalmed.pdf>. Accessed 18 Sept 2019.

14. VanderWeele TJ. A unification of mediation and interaction: a four-way decomposition. Epidemiology 2014;25(5):749-761.

15. Yung YF, Lamm M and Zhang W. Causal mediation analysis with the CAUSALMED procedure. <https://www.sas.com/content/dam/SAS/support/en/sas-global-forum-proceedings/2018/1991-2018.pdf>. Accessed 18 Sept 2019.

| **Supplementary Table 1** Description of participants included and not included in analyses. | | |  |
| --- | --- | --- | --- |
|  | Participants  included | Participants  not included | P-value |
|  |
| N | 204 | 479 |  |
| Age (years), mean±SD | 39.3 ± 16.9 | 38.6 ± 16.3 | 0.61 |
| Male, n (%) | 99 (48.5) | 263 (54.9) | 0.13 |
| Smoking status, n (%)  Never-smoker  Ex-smoker  Current smoker | 109 (53.4)  46 (22.6)  49 (24.0) | n=477  230 (48.2)  120 (25.2)  127 (26.6) | 0.46 |
| BMI (kg/m²), mean±SD | n=203  23.8 ± 3.84 | n=411  24.5 ± 4.51 | 0.05 |
| Socioprofessional category, n (%)  Unemployed  Manager  Technician  Manual worker | 32 (15.7)  64 (31.4)  88 (43.1)  20 (9.8) | n=474  63 (13.3)  147 (31.0)  199 (42.0)  65 (13.7) | 0.50 |
| Residence time (years), mean±SD | 11.7 ± 10.2 (0-42) | n=476  9.39 ± 0.74 (0-46) | 0.005 |
| Persistent asthma, n (%) | 161 (78.9) | n=151  128 (84.8) | 0.16 |
| Plasma FlOPs levels RFU/mL, GM (q1-q3) | 91.98 (79.47-104.69) | n=383  91.28 (77.32-191.86) | 0.71 |
| Air pollutants levels and traffic metrics^a^, mean±SD  NO_2_  NO_X_  PM_10_  PM_2.5_  Traffic load  Traffic intensity  O_3_  O_3-summer_ | 27.9 ± 13.9  49.7 ± 32.7  (n=146) 25.0 ± 4.07  (n=146) 15.0 ± 2.04  1755353 ± 4718630  7307 ± 13109  43.9 ± 4.62  65.4 ± 6.20 | (n=237) 30.6 ± 12.8  (n=237) 55.1 ± 28.2  (n=185) 25.3 ± 3.86  (n=185) 15.5 ± 1.93  (n= 237) 1703644 ± 4316804  (n=237) 7965 ± 14162  (n=445) 48.6 ± 8.30  (n=445) 67.5 ± 7.46 | 0.03  0.06  0.44  0.04  0.90  0.61  <0.0001  0.0007 |
| SD: standard deviation; BMI: body mass index; FlOPs: fluorescent oxidation products; GM: geometric mean; q1-q3: 25^th^ and 75^th^ percentiles of the GM.  ^a^Pollutant levels in µg/m^3^, traffic load in vehicles/day x meters, and traffic intensity in vehicles/day. | | | |

| **Supplementary Table 2** Associations between plasma FlOPs levels and characteristics of participants. | | | | | |
| --- | --- | --- | --- | --- | --- |
|  | n | GM | q1 | q3 | p-value*^a^* |
| Age  16-25  25-35  35-45  45-55  ≥55 | 67  33  17  44  43 | 86.6  87.1  83.0  99.1  101.6 | 76.1  75.7  79.6  84.1  86.2 | 94.5  102.7  88.4  110.4  111.2 | 0.0003  <0.0001^b^ |
| Sex  Men  Women | 99  105 | 90.6  93.3 | 77.6  79.6 | 102.7  106.1 | 0.19 |
| Smoking status  Never smoker  Ex-smoker  Current smoker | 109  46  49 | 88.3  98.2  94.6 | 76.6  84.1  81.4 | 97.41  111.2  104.8 | 0.02 |
| BMI kg/m²  <20  20-25  25-30  ≥30 | 30  104  58  11 | 87.8  92.8  93.11  87.3 | 76.1  79.7  79.6  73.6 | 96.6  104.9  104.8  102.5 | 0.33 |
| Socioprofessional category  Unemployed  Manager  Technician  Manual worker | 32  64  88  20 | 83.4  98.4  92.5  84.5 | 71.9  82.7  79.7  75.9 | 92.1  108.3  105.2  96.07 | 0.12 |
| BMI: body mass index; FlOPs: fluorescent oxidation products; GM: geometric means of FlOPs; q1-q3: 25^th^-75^th^ percentiles of the GM.  ^a^Adjusted for, age, sex and smoking status.  ^b^Trend test. | | | | | |

| **Supplementary Table 3** Associations between outdoor air pollution and persistent asthma. | | | | | | | | | |
| --- | --- | --- | --- | --- | --- | --- | --- | --- | --- |
|  |  | NO_2_ | NO_x_ | PM_10_^a^ | PM_2.5_^a^ | Total traffic load on major roads in a 100-m buffer of the home^b^ | Traffic intensity at the road nearest to a participant’s home^b^ | O_3_ | O_3-summer_ |
| Model 1 | n  OR crude  (95% CI) | 204  0.89  (0.69, 1.15) | 204  0.93  (0.75, 1.15) | 186  0.92  (0.30, 2.80) | 186  0.53  (0.18, 1.57) | 204  1.03  (0.74, 1.43) | 204  1.02  (0.88, 1.18) | 204  1.76  (0.75, 4.14) | 204  1.26  (0.69, 2.28) |
| Model 2 | n  OR adjusted  (95% CI) | 204  0.90  (0.69, 1.17) | 204  0.94  (0.75, 1.17) | 186  1.09  (0.35, 3.40) | 186  0.62  (0.20, 1.92) | 204  1.04  (0.74, 1.45) | 204  1.04  (0.89, 1.22) | 204  1.78  (0.73, 4.37) | 204  1.18  (0.63, 2.21) |
| Model 3 | n  OR adjusted  (95% CI) | 204  0.90  (0.71, 1.15) | 204  0.94  (0.77, 1.14) | 72  1.30  (0.48, 3.48)^c^ |  |  |  |  |  |
| Model 4 | n  OR adjusted  (95% CI) | 186  0.92  (0.69, 1.22) | 186  0.96  (0.75, 1.23) | 133  0.96  (0.27, 3.40) | 133  0.63  (0.19, 2.03) | 186  1.02  (0.71, 1.45) | 186  1.05  (0.89, 1.24) | 186  1.81  (0.68, 4.87) | 186  1.42  (0.73, 2.76) |
| Participants with remittent asthma were used as reference. The logistic models were conducted with random effects on familial dependence and center. NO_2_, nitrogen dioxide; NO_x_, nitrogen oxides; PM_10_, particulate matter with a diameter ≤10 µm; PM_2.5_, particulate matter with a diameter ≤5 µm. Results are expressed per 20 µg/m^3^ increase of NO_x_ exposure, per 10 µg/m^3^ increase of NO_2_, PM_10_, O_3_ and O_3-summer_ exposures, per 5 µg/m^3^ increase of PM_2.5_, per 4 million vehicles x meters per day increase of total traffic load, per 5 000 vehicles per day increase of traffic intensity. Model 1: unadjusted. Model 2: adjusted for age, sex and smoking status. Model 3: with back-extrapolated pollution and adjusted for age, sex and smoking status. Model 4: after excluding the participants living at the same residential address < 1 year;  ^a^Not back-extrapolated PM were only estimated in Paris and in Grenoble, and back extrapolated PM_10_ only in Paris.  ^b^Estimates were also adjusted for background NO_2_.  ^c^The linear regression model was only conducted with random effects on familial dependence because back-extrapolated PM_10_ were estimated only in Paris | | | | | | | | | |

| **Supplementary Table 4** Results of mediation analysis using the CAUSALMED procedure among participants who lived at the same address for >1 year (n=186). | | |
| --- | --- | --- |
| OR | Estimate | 95% CI |
| Odds Ratio Total Effect | 2.40 | 0.60–17.4 |
| Odds Ratio Natural Direct Effect | 1.76 | 0.48–8.93 |
| Odds Ratio Natural Indirect Effect | 1.37 | 1.01–2.94 |
| **Decomposition of the total effect** | | |
| Four-way | Percent | |
| Controlled direct | 43.9 | |
| Reference interaction | 10.0 | |
| Mediated interaction | 32.6 | |
| Pure indirect | 13.5 | |
| FlOPs: fluorescent oxidation products. Models were adjusted for age, sex and smoking habits. The exposure was O_3_. The mediator were plasma FlOPs levels. The outcome was persistent asthma. | | |

| **Supplementary Table 5** Controlled direct effect according to quantiles of plasma FlOPs levels (n=204). | | | | | |
| --- | --- | --- | --- | --- | --- |
| Quantiles of plasma FlOPs levels | 10%  (1.85 RFU/mL) | 25%  (1.90 RFU/mL) | 50%  (1.95 RFU/mL) | 75%  (2.02 RFU/mL) | 90%  (2.07 RFU/mL) |
| Controlled direct effect OR (95%CI) | 1.21 (0.32, 5.00) | 1.36 (0.45, 3.51) | 1.54 (0.62, 3.62) | 1.80 (0.63, 6.78) | 2.05 (0.53, 12.3) |
| FlOPs: fluorescent oxidation products. Participants with remittent asthma were used as reference. Results are expressed per 10 µg/m^3^ increase of O_3_. Models were adjusted for age, sex and smoking status. | | | | | |

**Figure legend.**

Supplementary Figure 1. Flow chart of the studied population.

FlOPs: fluorescent oxidation products
