# Supplementary figures and images for: Does the oxidative stress play a role in the associations between outdoor air pollution and persistent asthma in adults? Findings from the EGEA study
Source: Environ Health. 2019 Oct 29;18:90. doi: 10.1186/s12940-019-0532-0 (PMC6819357; doi:10.1186/s12940-019-0532-0)

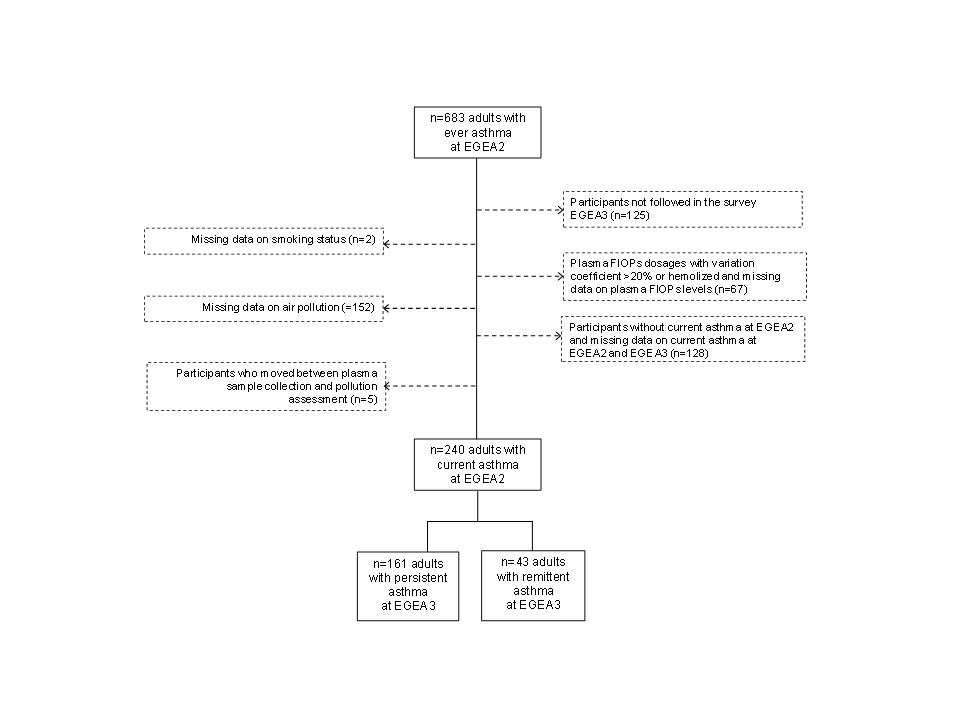

Supplement: Supplementary file 2 — Additional file 2: Figure S1. Flow chart of the studied population. [file 12940_2019_532_MOESM2_ESM.tif]
